# Selective Wet Etching for Scalable Nanofabrication of Patterned MXene Thin Films

**DOI:** 10.1021/acs.nanolett.5c02975

**Published:** 2025-09-15

**Authors:** Bar Favelukis, Barak Ratzker, Yonatan Juhl, Noy Stein Chneider, Omer Ashuach, Avia Greenberg, Jürgen Jopp, Pini Shekhter, Maxim Sokol

**Affiliations:** † Department of Materials Science and Engineering, 26745 Tel Aviv University, P.O.B 39040, Ramat, Aviv 6997801, Israel; ‡ Ilse Katz Institute for Nanoscale Science and Technology, Ben-Gurion University of the Negev, P.O.B 653, Beer-Sheva 8410501, Israel; § Tel Aviv University Center for Nanoscience and Nanotechnology, Tel Aviv University, P.O.B 39040, Ramat, Aviv 6997801, Israel

**Keywords:** MXene, Wet etching, H_2_O_2_, Micropatterning

## Abstract

The integration of MXenes, a class of conductive two-dimensional materials, into the microelectronics industry is largely hindered by the lack of scalable patterning methods. Herein, we present a novel wet etching approach for Ti_3_C_2_T_*z*_ MXene micropatterning, offering a facile, clean, cost-effective, and highly controllable technique that preserves the MXene intrinsic electrical and structural properties. By tailoring the etching solution and process parameters, micropatterned MXene electrodes with ∼200 nm lateral resolution were produced. The patterned films were applied to functional devices, including metal–semiconductor−metal (MSM) photodetectors, which demonstrated high conductivity and enhanced photoresponsivity. This work represents the first demonstration of wet etching as a viable method for highly precise MXene patterning, providing a scalable solution for next-generation MXene-based microelectronic technologies.

## Graphical abstract

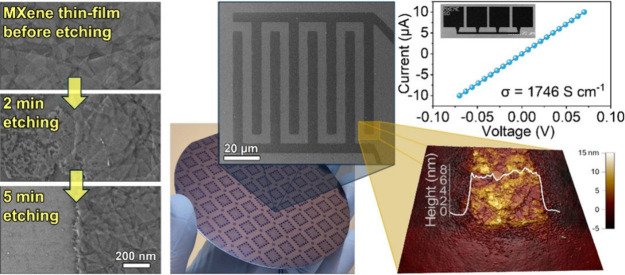

Materials development is typically the bottleneck for device miniaturization in the microelectronics industry.^1^ As dimensions approach the few-nanometer scale, the conductivity of common metallic interconnects and electrodes becomes limited due to surface scattering and electromigration.^2^ Therefore, shifting the focus toward alternative lower-dimensional materials.^3^ Materials such as CNTs, graphene, and TMDs are explored as alternative interconnects, electrodes, and transistor channels due to their superior physical properties. However, integrating these 2D materials into modern technologies is challenging, involving arduous fabrication processes.^4–6^

MXenes, a recent class of 2D materials, combine outstanding physical properties with promising synthesis scalability.^7^ These 2D transition metal carbides, nitrides, or carbonitrides–exhibit excellent metallic conductivity, high breakdown current density, chemical stability, hydrophilicity, and mechanical strength.^8^ MXenes are typically synthesized by etching precursor layered MAX phases to remove the A layer, using an HF-based solution or Lewis acid molten salt.^9^ They are converted into their 2D form by intercalation and exfoliation into a colloidal solution. Their general formula is M_n+1_X_n_T_*z*_ (n = 1–4), where M is an early transition metal, X is carbon and/or nitrogen, and T_*z*_ represents surface termination groups, often −O, −OH, −Cl, or −F.^10,11^ These surface terminations, determined by the etching process, significantly influences electronic properties, enabling a tunable work function from 1.6 to 6.2 eV.^12^

One of the key advantages of MXene is their synthesis and processability in colloidal solutions,^7,13^ enabling deposition at low temperatures and ambient conditions.^3^ Traditional micropatterning of sub-20 nm MXene features relies on lift-off methods, where MXene is first deposited onto a photoresist layer using techniques like spray coating, dip coating, or spin coating.^14,15^ Excess MXene is then removed by dissolving the underlying photoresist, leaving the desired pattern. While effective, this approach often leads to vertically oriented, folded flakes along pattern edges, resulting in inconsistent film thickness and poor lateral resolution.^14,15^ To date, the most reliable alternative at this scale is reactive ion etching (RIE), whereby the desired pattern is protected by photoresist while excess MXene is etched with CF_4_ (or other reactive) plasma.^16^ The main disadvantage of MXene patterning by RIE is the need for expensive equipment, the use of hazardous gases, and the lack of etching selectivity for different substrates.

In this work, we present the first demonstration of MXene thin-film patterning in ambient conditions using a wet etching process. By employing a well-known MXene pinhole etchant, we developed a selective etching process for MXene features with a lateral resolution down to 200 nm and a calibrated undercut etching rate, which can be easily performed with relatively simple tools and chemicals. Unlike the wet etching of other 2D materials like graphene^17,18^ and TMDs,^19,20^ herein we demonstrate wet etching of functional full-sized features on wafer scale.

## Etching of Ti_3_C_2_T_*z*_ Freestanding Films and Depostied Thin Films

The principle of etching MXene to create holey surfaces has been reported in several studies,^21–24^ typically by mixing diluted H_2_O_2_ with MXene colloidal solutions. MXene is known to be resistant to most acids but reacts with H_2_O_2_ to form TiO_*x*_ and amorphous carbon (Figure 1a). The edges are the most vulnerable areas in the MXene flakes and react rapidly with the H_2_O_2_ solution.^25^ Concurrently, pinholes form at surface defects, such as vacancies, creating more exposed edges being dissolved and accelerating the etching rate. In this work, we harness this phenomenon to pattern MXene thin films. First, we tested several mixtures of 1:3 H_2_O_2_ and different concentrated acids (i.e., sulfuric (H_2_SO_4_), nitric (HNO_3_), and hydrochloric (HCl) acids), demonstrating highly controllable etching of MXene freestanding films.

**Figure 1:**
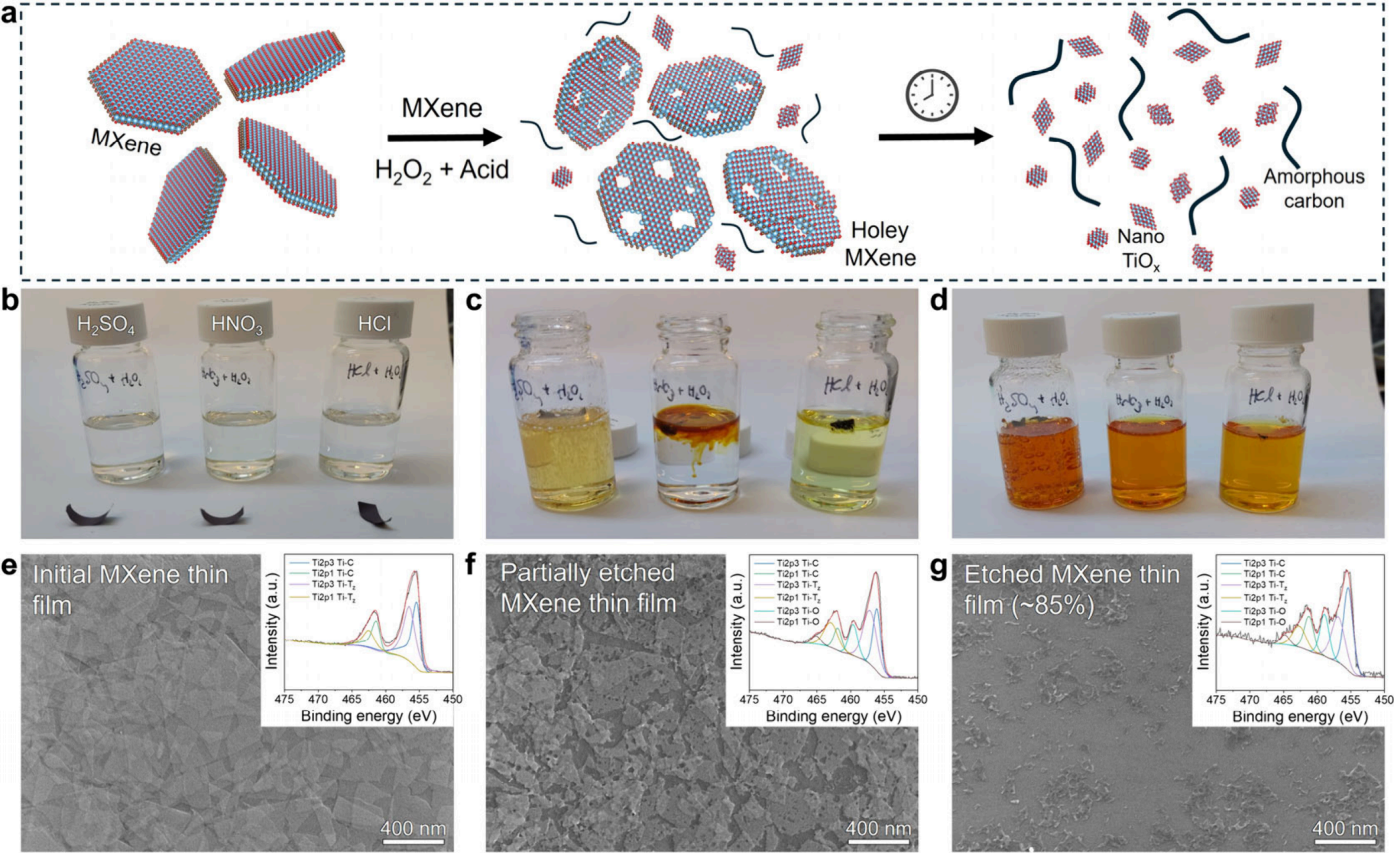
**MXene etching process. a** Schematic of MXene etching stages. **b–d** MXene free-standing film before, during, and after etching with H_2_O_2_ and different acids (H_2_SO_4_, HNO_3_, and HCl) in a 1:3 ratio. **e–g** SEM micrographs of MXene thin film before, during, and after etching; inset graphs show the respective Ti 2p XPS spectra.

The etched MXene byproducts dissolve in the solution as amorphous carbon and colloidal TiO_*x*_ nanoparticles, which impart an orange color to the solution, due to hydrogen occupying oxygen vacancies,^26,27^ as shown in Figure 1b-d. The XRD patterns of the initial MXene freestanding film and dried etched byproducts are given in Figure S1, showing a transition from the layered MXene structure to TiO_*x*_ (anatase) nanoparticles and amorphous carbon with some residual crystalline MXene. Similar results were reported by Jiang et al.,^21^ after using H_2_O_2_ to prepare MXene-based heterostructure catalysts. The H_2_O_2_–HCl solution was chosen for patterning the MXene thin films due to the stability of common commercial photoresists in HCl, allowing to etch at high concentrations.

Figure 1e-g shows SEM micrographs of 8 nm-thick MXene thin film deposited by spin coating on a silicon wafer before etching, after 2, and 5 min etching. Corresponding X-ray photoelectron spectroscopy (XPS) Ti 2p spectra are inserted in Figure 1e–g. The XPS measurements reveal that after etching of the MXene flakes most of the Ti remains unoxidized^28^ (full XPS spectra given in Figure S2). Zhang et al.^22^ showed that during the etching process H_2_O_2_ attacks the Ti sites on MXene surfaces – oxidizing it into TiO_*x*_ nanoparticles that are washed away into the solution, leaving behind intact porous MXene. It is likely that some portion of the observed Ti–O bonds in our XPS spectra correspond to unwashed residual TiO_*x*_ nanoparticles. We observed that even after relatively long etching time (>5 min) small areas of unetched MXene still remained, which were not removed by further etching duration. This critical issue was resolved by using a surfactant (Triton X-100) to increase surface tension and reduce the size of gas bubbles adhering to the MXene surface (Figure S3a) and blocking the etchant. Ultimately, adding a surfactant to the etching solution is essential for producing clean, sharp MXene patterns (Figure S4).

## Ti_3_C_2_T_*z*_ MXene Micropatterning by Wet Etching

Controlled patterning of the MXene thin-film was achieved using traditional photolithography, as illustrated in Figure 2a-c. The photoresist covering the flake surface significantly decreased the etching rate of the film, thus allowing regulation primarily through single-edge etching. The MXene flake morphology after 0, 2, and 5 min of etching (Figure 2d-f) illustrates the etching mechanism: etching of uncovered MXene progresses through the flake edges coupled with pinholes forming new edges in the basal planes. The individual flakes etch gradually, resulting in sequential etching of the MXene layers (Figure 2e). The etching eventually removes all the unprotected MXene while progressing homogeneously by the flake edges under the photoresist-protected area (Figure 2f). The resulting features exhibit an edge roughness of less than 100 nm, as shown in Figure 2f and Figures S5–S7.

**Figure 2:**
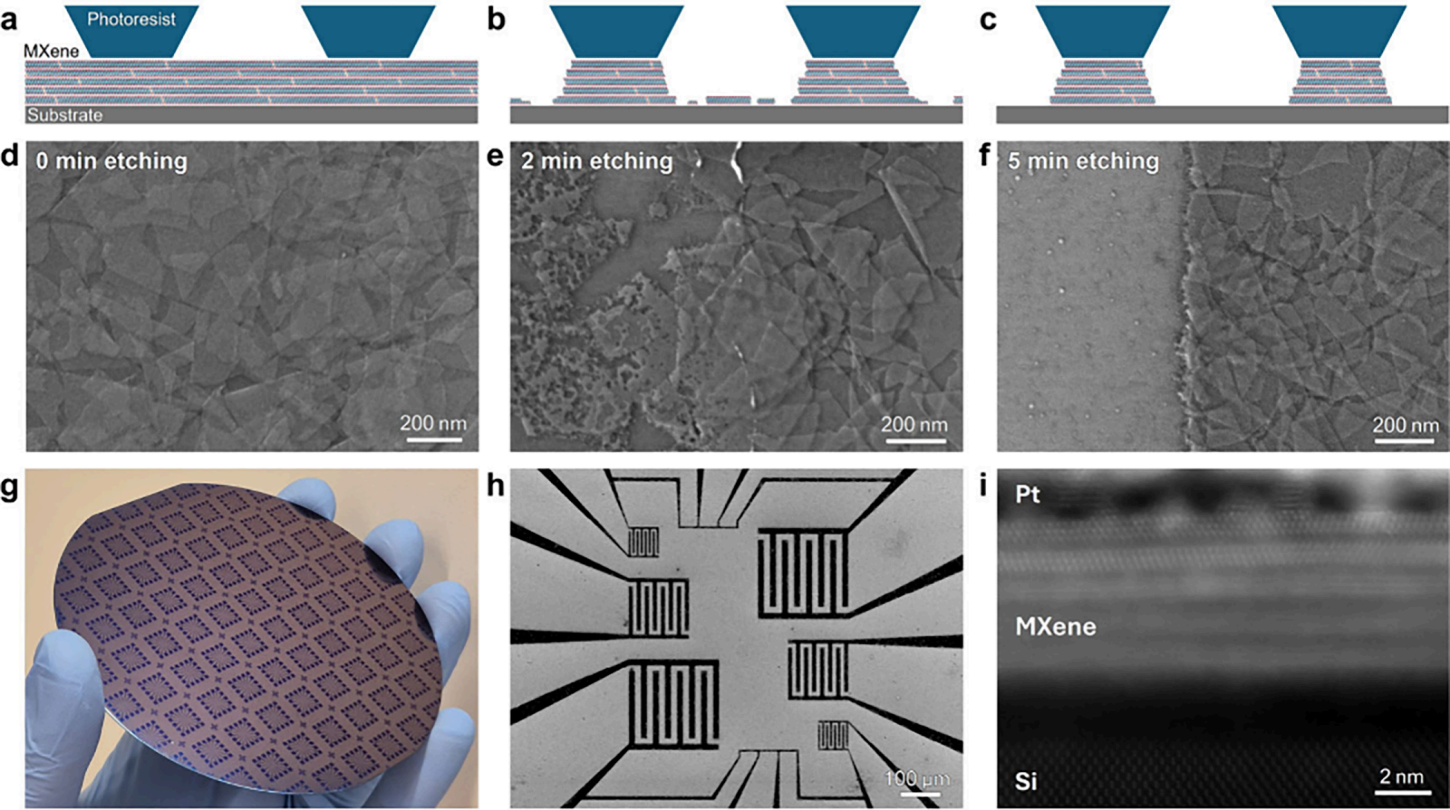
**MXene patterning using wet etching. a–c** Schematic of MXene thin film patterning process using wet etching. SEM micrographs of the **d** initial MXene thin-film, **e** after partial etching, and **f** at the end of the etching process (after removal of the photoresist). **g** MXene-coated 4” wafer patterned by the wet etching process. **h** SEM micrograph of the MXene-patterned electrodes. **i** High-resolution STEM micrograph of the MXene thin-film cross-section.

After MXene deposition residual water remains between the flakes, which desorbs during heating.^29,30^ Therefore, it is necessary to heat the MXene above the photoresist baking temperature to prevent bubble formation under the photoresist caused by water degassing (Figure S3b). Fortunately, Ti_3_C_2_T_*z*_ can withstand heating in an ambient atmosphere up to 245 °C without oxidation.^31^ The MXene surface is highly hydrophilic due to −O and −OH terminations, which can cause photoresist adhesion issues and uneven etching (Figure S3c). To solve this issue, photoresist adhesion to the MXene film was ensured by spin coating with hexamethyldisilazane (HMDS) followed by heating at 200 °C. HMDS reacts with the MXene terminations, adding imido and amido silicate complexes to the MXene terminations, as measured by XPS (Figure S8),^32^ improving photoresist adhesion.

A major advantage of wet etching approaches is that they are readily adaptable to wafer scale. Figure 2g shows a 4-in. silicon oxide wafer with a high yield of 1 × 1 cm^2^ dies with well-defined, sharp electrodes (Figure 2h). The process was also demonstrated on 4-in. fused silica, as shown in Figure S9. By the end of the process, the electrode surface remains undamaged, as shown in the SEM micrographs (Figure 2d-f and Figure S5), and in the cross-sectional TEM micrograph (Figure 2i). Thus, exhibiting the straightforward scalability potential of MXene patterning by wet etching using common techniques and materials in the microelectronics industry.

## Wet Etched Electrodes Characterization and Fabrication Limitations

MXene electrodes are commonly patterned by a lift-off process due to its simplicity. Therefore, we compare the wet etching to state-of-the-art patterening by lift-off^14,15^ performed on the same kind of deposited MXene thin-film and electrode design. The clear advantage of the wet etching process can be seen in comparative thickness profiles obtained by AFM cross-sectional scans (Figure 3a) as well as the SEM micrographs of electrodes patterned by lift-off^14,15^ (Figure 3b) and wet etching (Figure 3c). It can be clearly seen that the wet-etched electrodes exhibit dramatic improvement in lateral resolution and thickness uniformity. Moreover, while the lateral resolution of MXene thin films patterned by lift-off is fundamentally limited by the average flake size,^15^ there is no such constraint when using a wet etching approach. This is a major benefit as having a larger MXene flakes significantly enhances the electrical conductivity.^33^ Further AFM scans are provided in Figure S10, showing that the electrodes comprise pristine MXene flakes that were protected by the photoresist during etching. AFM thickness profiles (Figure 3d-f) and their corresponding area scans (Figure 3g-i) are shown for different-sized electrodes and average height profile in an overetched sample is shown in Figure 3d-i. The average edge profile reveals a stepped structure corresponding to the number of MXene flakes at the smallest electrode marked by orange arrows (in Figure 3d), which becomes less prominent in the wider electrodes, leading to nearly vertical edges.

**Figure 3:**
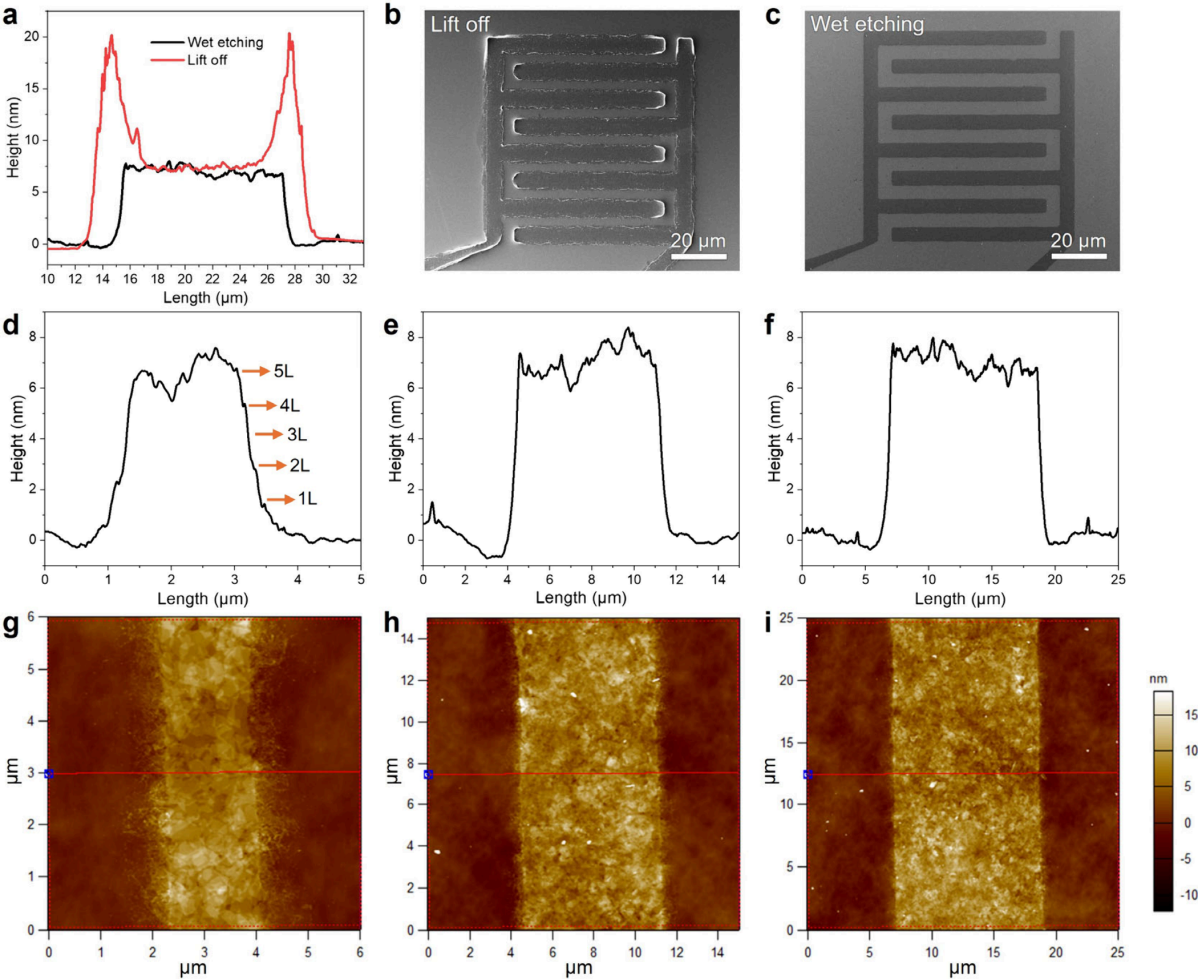
**AFM analysis of patterned MXene electrodes. a** AFM average height profile of MXene electrodes patterned by lift-off and wet etching. Corresponding SEM micrographs of the entire MXene electrodes patterned by **b** lift-off and **c** wet etching. **d–f** AFM average height profiles of MXene electrodes with different widths and **g–h** corresponding AFM scans.

Contrary to the hitherto micropatterning methods applied for MXenes, such as dry etching, lift-off, and microcontact printing that require expensive equipment, specialized environments, or suffer from limited resolution and reproducibility, the wet-etch process offers a facile, clean, cost-effective, and highly controllable technique that preserves the MXene intrinsic electrical and structural properties. The process avoids the use of high temperatures and harmful reagents and can be easily performed with relatively simple tools and chemicals on various substrates to afford functional MXene electrodes on a full-sized wafer scale. The comparative characteristics of different MXene thin-film micropatterning methods are presented in Table 1. The wet etching process offers several advantages, combining excellent lateral resolution with high selectivity, throughput and scalability, as well as short processing time and low cost (both in reagents and equipment) under ambient conditions. Notably, wet etching yields precisely patterned MXene with lateral resolution on par with RIE, while using much simpler and cost-effective means. Furthermore, wet etching offers significantly higher selectivity as H_2_O_2_ solutions are inert toward common substrates (e.g., Si, SiO_2_, SiC, sapphire), unlike the aggressive F- and Cl-based plasma used in RIE that can readily etch the substrate material.

**Table 1:** Comparison between MXene Micropatterning Methods

Patterning method	Lateral resolution	Processing time	Thickness uniformity	Selectivity	Scalability^a^	Reagents and equipment requirements	Cost
Wet etching	200 nm	2–8 min	0.5–0.8 nm	High	High	Low	Low
RIE^16,34^	100–200 nm	10–40 min	0.5–2 nm	Low	High	High	High
Lift-off^15,35^	1.5–25 μm	1–30 min	10–60 nm	High	Medium	Low	Low
Contact printing^36,37^	5–300 μm	1–5 min	2–200 nm	High	Low	Low	Low

a Prediction based on technical feasibility; so far all photolithography techniques were demonstrated on 4” wafer scale.

The undercut etching rate of MXene films is crucial in integrated circuit fabrication processes, where etching must be precisely controlled to maintain feature dimensions and prevent excessive undercutting beneath other structures.^38^ The MXene undercut etching rate was calculated by etching different samples for 1–8 min (Figure S6) and measuring the electrode dimensions. The dimensions after etching for 4, 6, and 8 min can be seen in the SEM micrographs in Figure 4a-c. As with conventional isotropic etching, the electrode size decreases beneath the photoresist, rounding the corners of the features. This occurs due to the cumulative effect of multidirectional etching, which removes more material from the corners than from straight edges or flat surfaces, gradually rounding them over time. The undercut etching rate graph shown in Figure 4d starts after the electrodes are completely disconnected at 2 min of etching time. The undercut etching rate exhibits a linear behavior, which for the etching conditions employed herein can be expressed as *X­(t)* = 0.36*t* – 0.89, where *X* is the undercut length in μm and *t* is time in min. Complete removal of MXene residue from the uncovered surfaces is observed at 3 min (Figures S6, S7), theoretically making this the limit for ∼200 nm features under the employed etching conditions (i.e., 1:3 H_2_O_2_:HCl 0.5 vol % Triton X-100 at room temperature).

**Figure 4:**
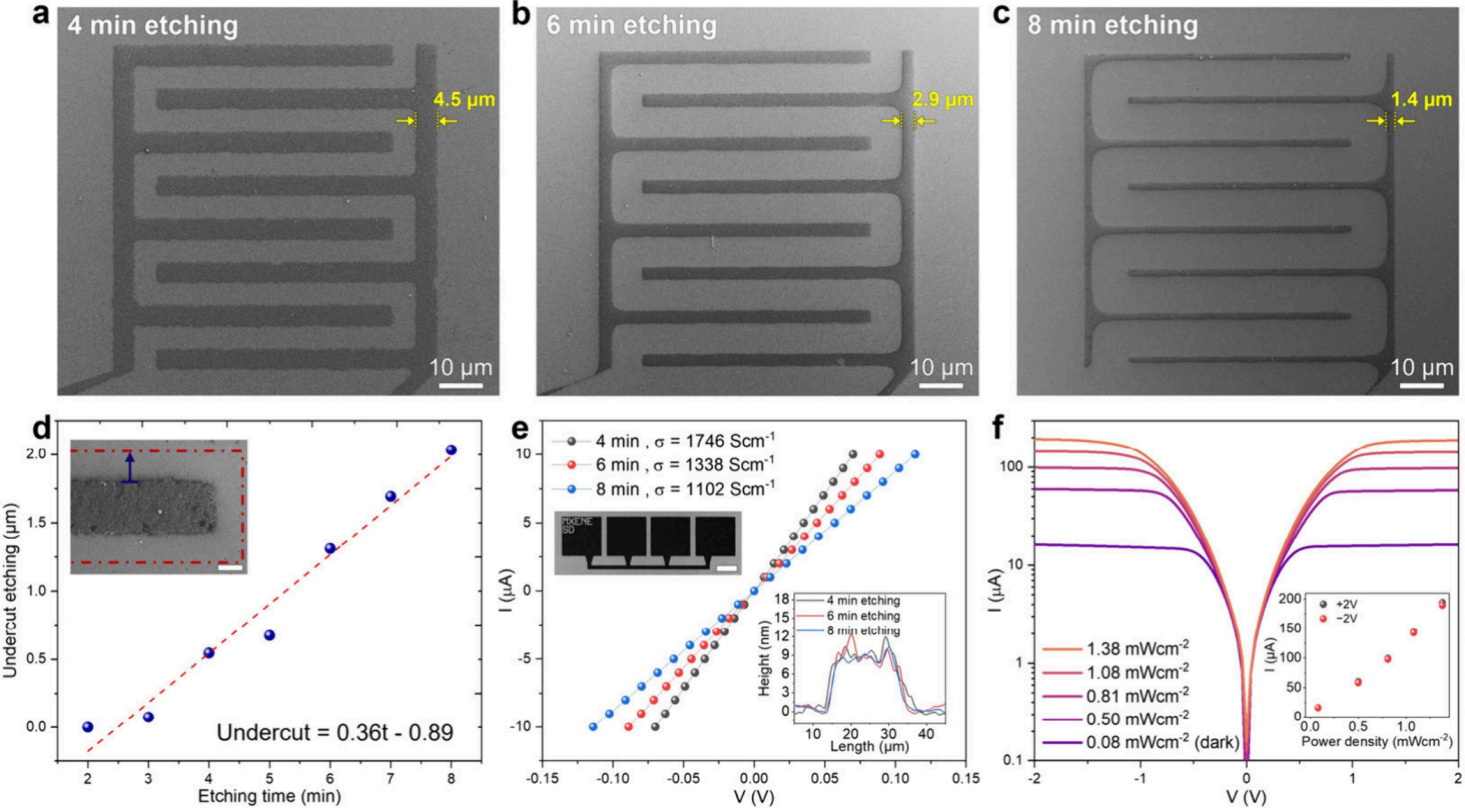
**MXene etching calibration and performance. a–c** MXene electrodes patterned by wet etching for 4, 6, and 8 min. **d** Undercut etching rates, with the inset showing an SEM micrograph of an overetched electrode (scale bar 1 μm). The blue arrow represents the undercut etching length. **e** Four-point probe measurement of MXene features at different etching times. The upper left inset shows an SEM micrograph of the MXene four-point electrode (scale bar 100 μm), and the bottom right graph shows the features height. **f** IV measurement of a MXene-Si-MXene photodetector, with the inset displaying photocurrent at ± 2 V versus illumination power density.

Improving the lateral resolution below ∼200 nm would require enhancing the surface (basal plane) etching while minimizing the flake edges etching. This could be achieved by fine-tuning the etching parameters. Conceivably, by adjusting the H_2_O_2_/acid solution concentration and etching temperature, or finding an alternative etchant with basal plane selectivity. Additionally, control over the etching rate can vary between MXene synthesis types, flake sizes, and stoichiometries. Compared to TMDs and graphene, the common MXene synthesis involves aggressive chemical conditions, such as HF or molten salt Lewis acids and mechanical exfoliation, leading to a relatively high concentration of defects. The MXene surface defects are a key factor in the formation of pinholes,^39^ which increase the etching rate. Since etching progresses through the flake edges, the concentration of surface defects and flake size will affect the etching rate.^40^

Electrical properties were measured to assess the performance of the wet-etched MXene electrodes. The IV curve presented in Figure 4e shows the electrical conductivity of patterned MXene electrodes etched at 4, 6, and 8 min on SiO_2_, measured using a four-point probe configuration with an initial width of 20 μm, as shown in the upper inset of Figure 4e. The MXene electrode shows conductivity of 1102–1746 S·cm^–1^ typical to multiflaked MXene films.^41^ The profile measurement shown in the bottom inset of Figure 4e illustrates that although the electrode width decreases by approximately 20% between 4 and 8 min of etching, the electrical conductivity decreases by 37% S·cm^–1^. This nonlinear relationship is likely due to an inhomogeneous etching effect that becomes more apparent with extended etching times (as shown in the AFM scan in Figure 3g). MXene comb-shaped electrodes were patterned by 4 min etching on n-type Si to create MXene-Si-MXene (MSM) Schottky junction, which were tested as photodetectors. The IV curve shown in Figure 4f demonstrates that the MSM junctions exhibit high sensitivity to light across various illumination intensities, even under relatively low photon flux,^42^ with a responsivity of 11.03 A/W.

## Conclusions

This work demonstrates a novel patterning method for MXene electrodes, using a wet etching process. This method requires common nanofabrication tools and reagents operating at ambient conditions, simplifying the integration of MXene into the microelectronics industry. By harnessing the well-known MXene pinhole etchant (H_2_O_2_), we demonstrate highly controllable patterning of Ti_3_C_2_T_*z*_ MXene sub-10 nm thin-films on a variety of common substrates. MXene micropatterning is achievable with an excellent lateral resolution of ∼200 nm with the potential for further resolution improvement. The selective wet etching process creates sharp edge features without damaging the substrate and enables utilization of the undercut etching rate to fine-tune the final electrode width for desired applications. The patterned MXene electrodes exhibit high electrical conductivity when deposited on a dielectric substrate and function as a highly sensitive photodetector when deposited on Si. This wet etching approach paves the way for the large-scale integration of MXenes into next-generation microelectronic nanoscale devices.

## Methods

### MAX Phase Synthesis

Titanium carbide (TiC, 99.5%, Alfa Aesar), titanium (Ti, 99.7%, Strem), and aluminum (Al, 99.7%, Strem) powders were mixed in a 2:1:1.1 ratio and tumbler-mixed with stainless steel balls at 200 rpm for 18 h. The blended powders were compacted into a pellet under a pressure of 5 MPa at room temperature. The green body pellet was heat-treated under an argon atmosphere in a tube furnace at 1450 °C for 3 h, with a heating/cooling rate of 5 °C/min. The obtained Ti_3_AlC_2_ MAX phase pellet was ground and sieved to obtain a powder with a particle size of ≤ 20 μm.

### MXene Synthesis

Ti_3_C_2_T_*z*_ MXene was synthesized by selectively etching Ti_3_AlC_2_. One g of MAX powder was slowly added to 20 mL of 10.2 M HCl (32%, Bio-Lab) with 1.6 g of lithium fluoride (LiF, 99%, Strem) at 45 °C and stirred for 24 h. After etching, the mixture was centrifuged at 3500 rpm several times with DI water until the pH reached 6. To exfoliate the MXene, the suspension was sonicated in an ultrasonic bath for 30 min while being kept at 5 °C. After sonication, the suspension was centrifuged at 3500 rpm for 30 min to separate any unexfoliated sediment. The MXene colloidal solution was bubbled with N_2_ for 15 min and stored at 4 °C. 5 ml of the colloid was dried and weighed to determine the MXene concentration, which was approximately 14 g/L.

### MXene Thin-Film Deposition

MXene deposition was performed using the protocol showcased in our previous study.^15^ Multiple wafer substrates were piranha-treated using a 1:3 H_2_O_2_/H_2_SO_4_ solution, respectively, (H_2_SO_4_ 98%, SDFCL; H_2_O_2_ 35%, ThermoFisher Scientific) to increase hydrophilicity. Si (4”, n-type, p-doped, 1–100 Ω·cm, University Wafers), SiO_2_ (4”, n-type, 100 nm wet thermal oxide, University Wafers), and fused silica (4”, University Wafers) wafers were spin-coated with MXene diluted to 7 g/L at 2000 rpm for 1 min, followed by spin-cleaning with 0.5 M HCl at 2000 rpm for 1 min, resulting in an 8 nm-thick MXene film.

### MXene Wet Etching

First, the MXene-coated wafers were heated to 200 °C for 5 min to remove residual water from the MXene. They were then spin-coated with HMDS (99%, Sigma-Aldrich) at 3000 rpm for 40 s and heated again to 200 °C for 5 min. The MXene electrode was patterned by photolithography; the substrate was spin-coated with Futurrex NR-1500PY negative photoresist at 3000 rpm for 40 s, baked at 150 °C for 1 min, exposed with 285 mJ/cm^2^ using SUSS MA6 as a mask aligner, postbaked at 100 °C for 1 min, and developed in RD6 solution for 17 s. For process calibration, some of the wafers were diced using a Disco DAD300 dicing saw.

MXene removal by wet etching was done using a 1:3 H_2_O_2_/HCl solution with 0.5 vol % Triton X-100 for different etching times. After etching, the photoresist was removed using acetone, IPA, and water.

### Characterization

X-ray diffraction (XRD) measurements of the freestanding MXene film and the etched byproducts were performed over a 2θ range of 3–85° with 0.02° steps using a Malvern Panalytical Aeris diffractometer. XPS analysis was carried out with an ESCALAB QXi (Thermo Scientific). The morphology of the MXene electrode was examined via high-resolution SEM using a ZEISS Gemini 300. AFM measurements were performed using the MFP-3D-Bio system (Asylum Research/Oxford Instruments) with an Olympus AC160TS probe (nominal *f*_0_ = 300 kHz, *k* = 26 N/m). The scans were taken in AC mode with a free amplitude (*A*_0_) set to 2.34 V (200 nm) and a set point amplitude (*A*_*s*_) of 1.423 V (125 nm), approximately 63% of *A*_0_. STEM imaging of the MXene thin-film cross-section was performed with a probe Cs-corrected (S-CORR) Thermo Fisher Spectra 200 operating at 200 kV. The lamella for STEM analysis was prepared using a dual-beam focused ion beam (FIB; FEI Helios G4 UC).

Electrical measurements were carried out with a Keithley 4200A I–V/C-V probe system. Electrical conductivity was measured using a 4-point probe configuration by applying currents of 1–10 μA and recording the induced voltage. The performance of the MSM photodiode was analyzed with a 2-point probe configuration, using 0.04 V step increments under varying white LED illuminations ranging from 0.08 to 1.38 mW/cm^2^. The undercut etching rate was calculated through image analysis using ImageJ software.

## Supplementary Material



## Notes

Additional characterization data of MXene films and micropatterned electrodes (PDF)
