# Supplementary material for: Selective Wet Etching for Scalable Nanofabrication of Patterned MXene Thin Films
Source: Nano Lett. 2025 Sep 15;25(38):14017–24. doi: 10.1021/acs.nanolett.5c02975 (PMC12464998; doi:10.1021/acs.nanolett.5c02975)
Supplement: Supplementary file 1 [file nl5c02975_si_001.pdf]

Supplementary Information

Selective Wet-Etching for Scalable Nanofabrication of Patterned MXene Thin Films

Bar Favelukis[1], Barak Ratzker[1], Yonatan Juhl[1], Noy Stein Chneider[1], Omer Ashuach[1], Avia Greenberg[1], Jürgen Jopp[2], Pini Shekhter[3], Maxim Sokol[1,*]

[1] Department of Materials Science and Engineering, Tel Aviv University, P.O.B 39040, Ramat Aviv 6997801, Israel
[2] Ilse Katz Institute for Nanoscale Science and Technology, Ben-Gurion University of the Negev, P.O.B 653 Beer-Sheva 8410501, Israel
[3] Tel Aviv University Center for Nanoscience and Nanotechnology, Tel Aviv University, P.O.B 39040, Ramat Aviv 6997801, Israel

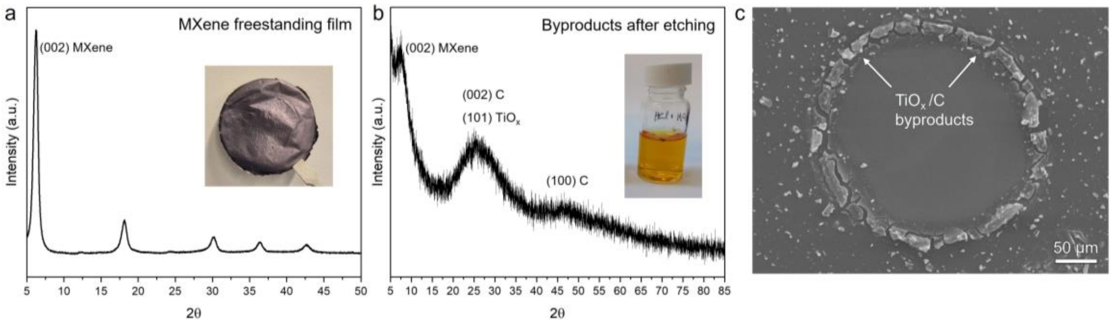

Figure S1. XRD patterns of **a** $Ti_3C_2T_z$ freestanding film, the inset shows a photograph of the film. **b** Residues of the $Ti_3C_2T_z/TiO_x/C$ byproducts after dissolving $Ti_3C_2T_z$ in H_2O_2 and HCl, inset shows a photograph of the solution. Most of the material is amorphous but weak peaks related to residual MXene and TiO_x (anatase) can be detected; note the signal-to-noise ratio is very low. **c** SEM micrograph of dried-up TiO_x/C residues.

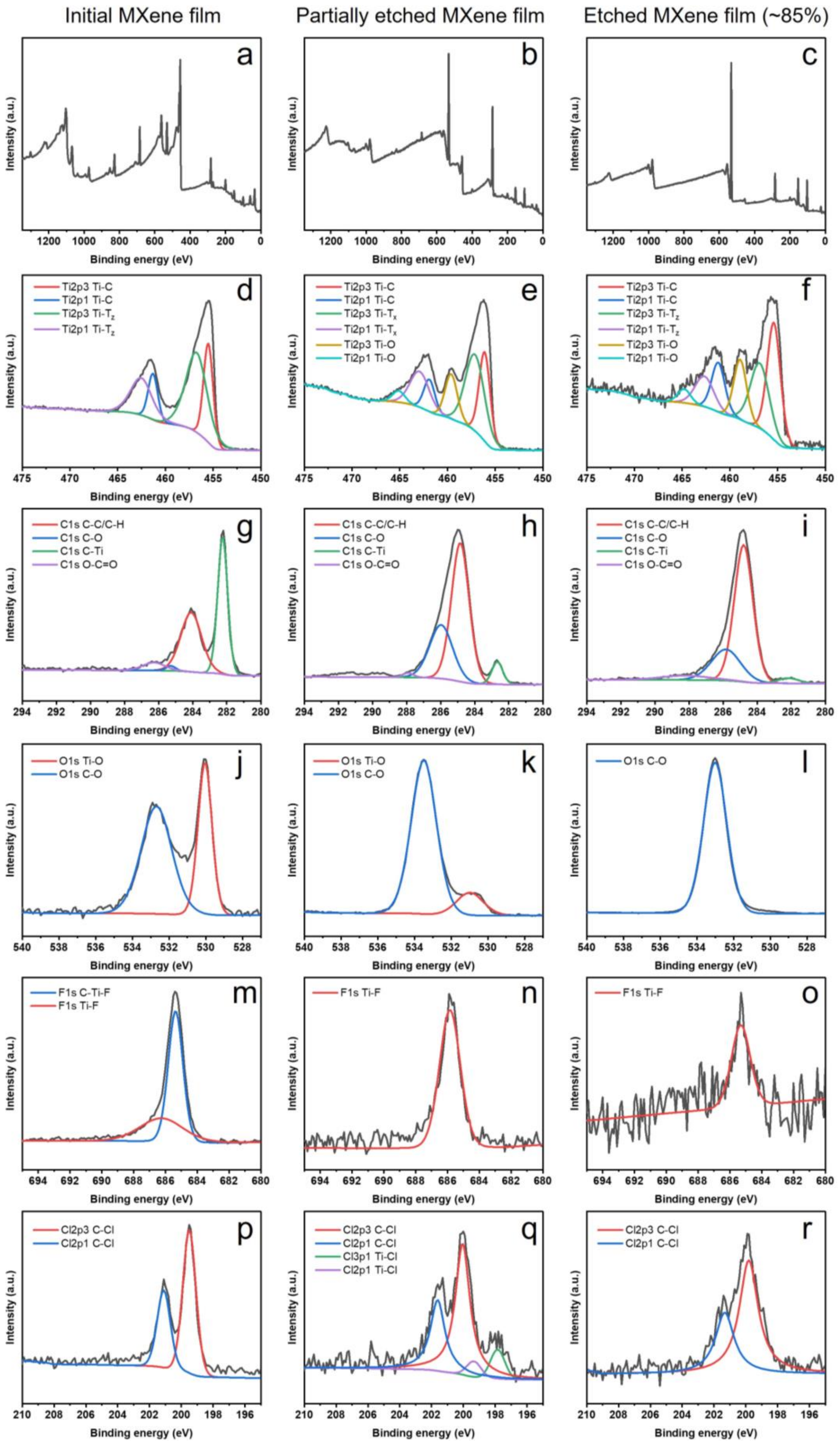

Figure S2. XPS spectra of pristine, partially and ~85% etched MXene thin-film on Si. **a-c** survey spectrum, **d-f** Ti 2p, **g-i** C 1s, **j-l** O 1s, **m-o** F 1s, p-r Cl 2p.

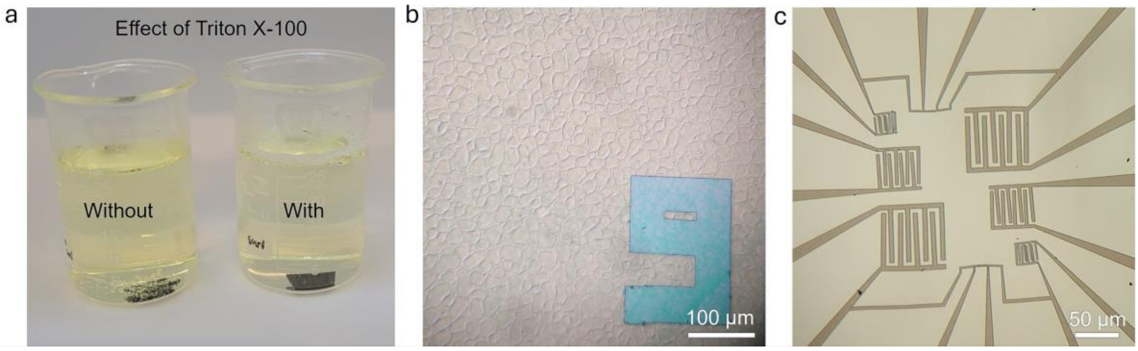

Figure S3. **a** Photograph of MXene electrodes being etched with (right) and without (left) Triton X-100. Optical micrographs showing **b** non-heat-treated MXene degassing under developed photoresist and **c** Adhesion problem of developed photoresist on MXene without HMDS.

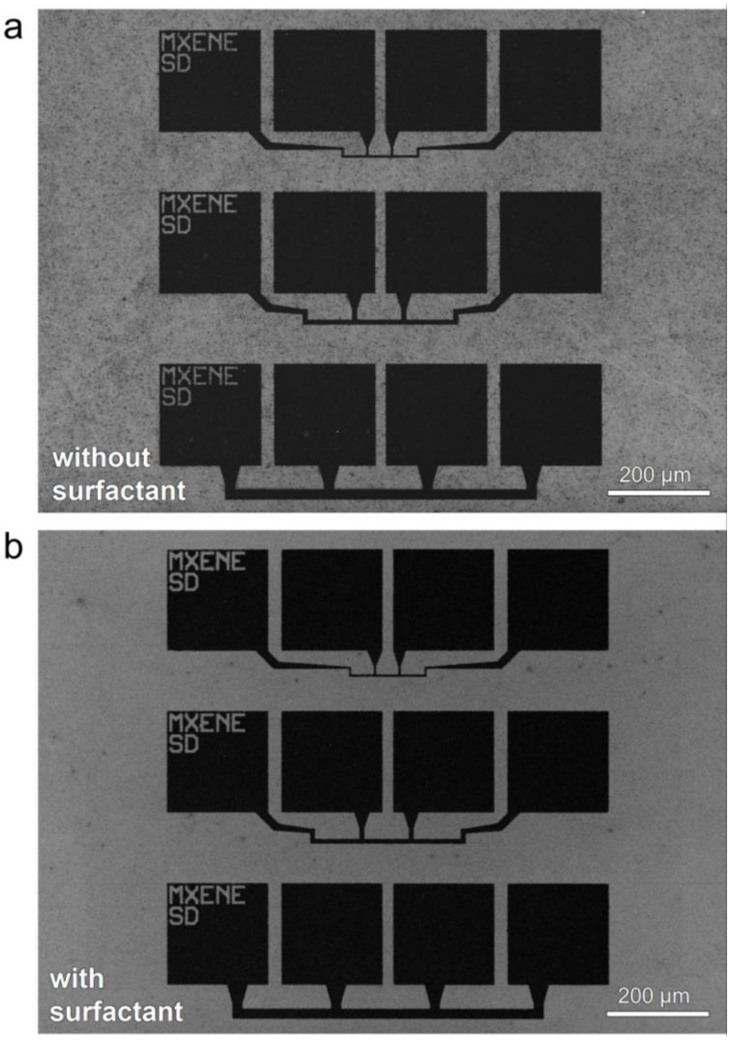

Figure S4. SEM micrographs of a MXene thin-film electrode patterned by 4-min wet etching **a** without and **b** with 0.5% vol of Triton X-100.

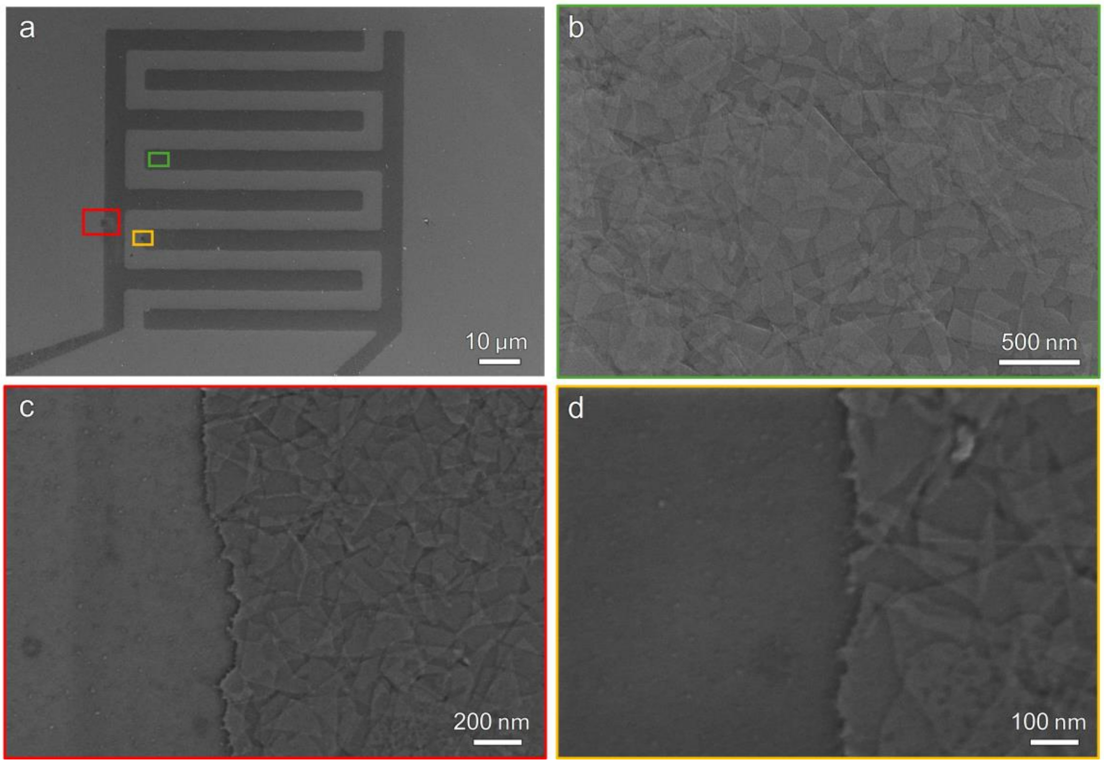

Figure S5. SEM micrographs of a MXene thin-film electrode patterned by 4-min wet etching. **a** low magnification of the electrode, with the colored rectangle indicating the high-magnification locations in **b-d**. The high-resolution SEM analysis showcases the high-quality MXene thin-films, patterning precision, and undamaged Si substrate.

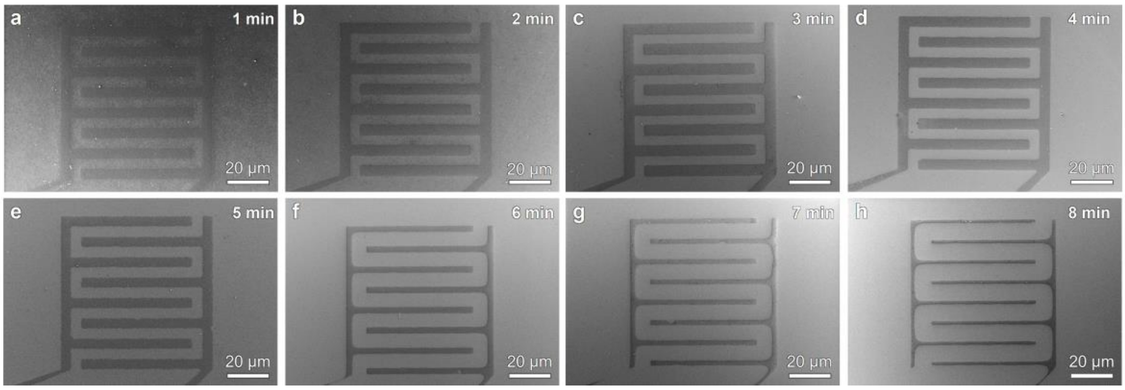

Figure S6. MXene electrodes patterned by wet etching at different etching times. The effect of undercutting progressing below the photoresist with extended etching durations is evident.

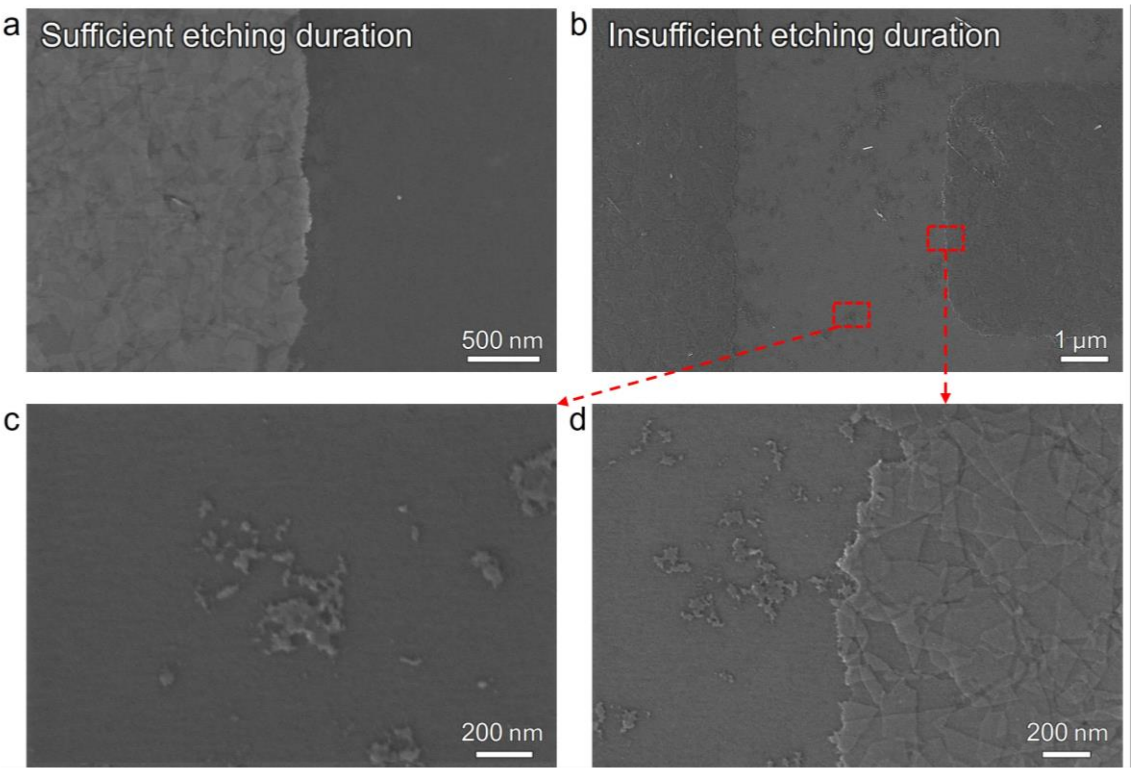

Figure S7. SEM micrographs of MXene wet etching stages before and after completion. **a** complete etching after 3 min, **b** incomplete etching after 2 min, **c** high magnification of MXene residues, **d** high magnification of electrode edge.

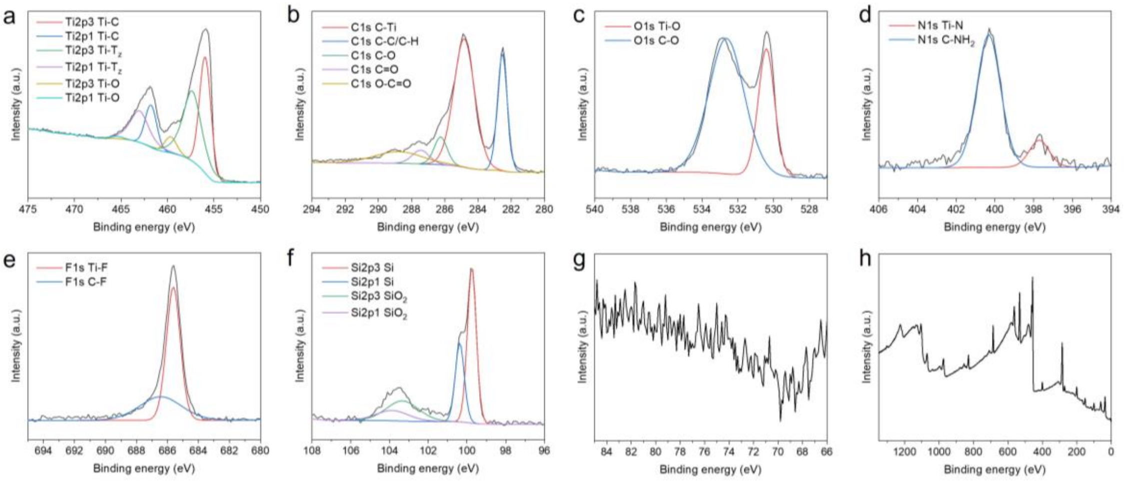

Figure S8. XPS spectra of HMDS-treated wet-etched MXene thin-film on Si. **a** Ti 2p, **b** C 1s, **c,** O 1s, **d** N 1s, **e** F 1s, **f** Si 2p, **g** Al 2p, **h** survey spectrum.

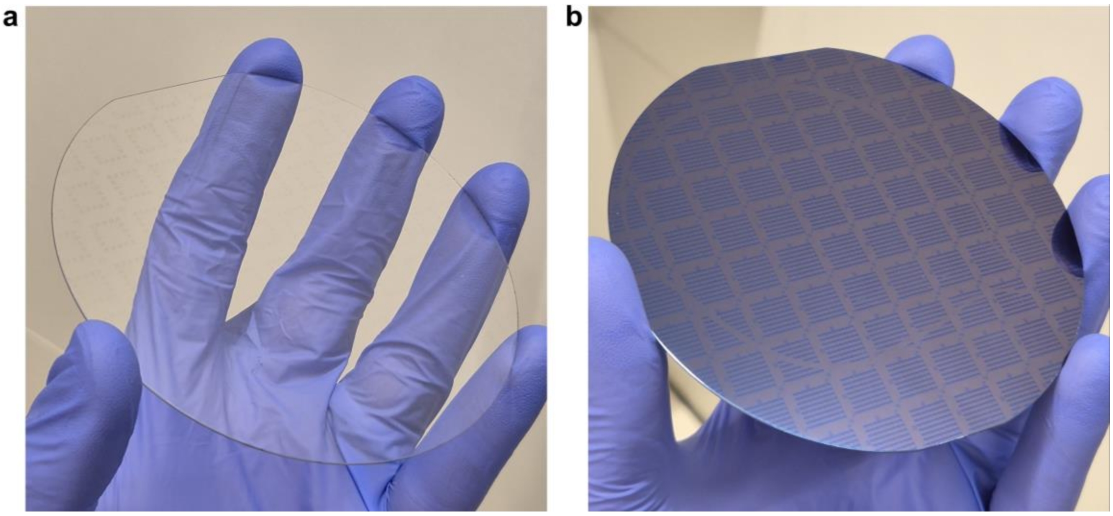

Figure S9. MXene electrode patterned by wet etching on a 4-inch wafer: **a** fused silica and **b** 100 nm SiO_2.

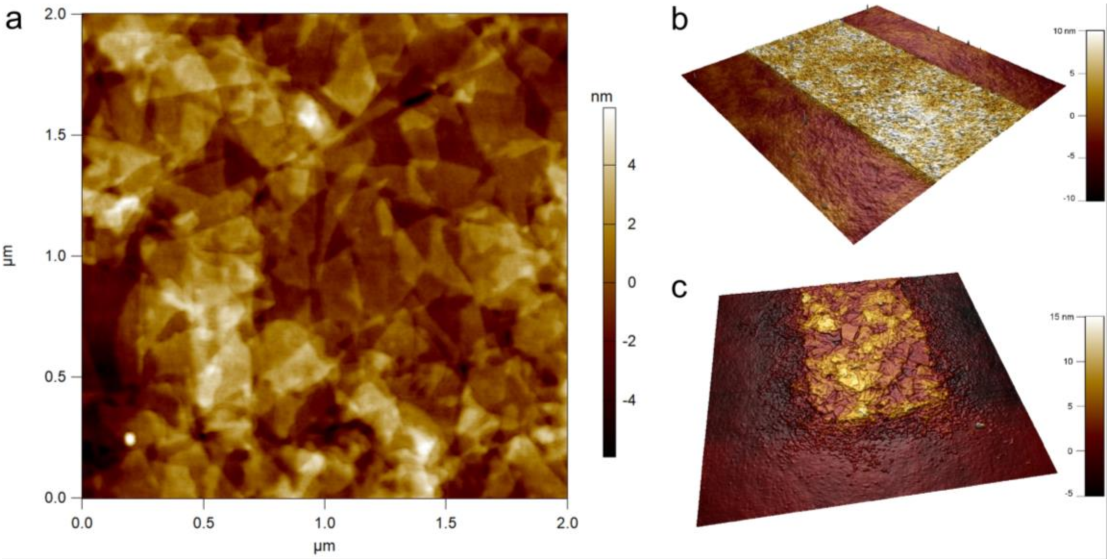

Figure S10. AFM scans of wet-etched MXene electrodes, **a** MXene thin film that was protected by photoresist, **b** 10 µm, and **c** 5 µm electrodes. Note that the MXene remains completely intact
